# Photo Phenosizer, a rapid machine learning-based method to measure cell dimensions in fission yeast

**DOI:** 10.17912/micropub.biology.000620

**Published:** 2022-08-04

**Authors:** Martin Vo, Lance Kuo-Esser, Mauricio Dominguez, Hayley Barta, Meghan Graber, Alex Rausenberger, Ryan Miller, Nathan Sommer, Wilber Escorcia

**Affiliations:** 1 Biology Department, Xavier University; 2 Lake Erie College of Osteopathic Medicine, Erie; 3 Math Department, Xavier University; 4 Department of Mathematics and Statistics, Grinnell College; 5 Computer Science Department, Xavier University

## Abstract

Cell metrics such as area, length, and width provide informative data about cell cycle dynamics. Factors that affect these dimensions include environmental conditions and genotypic differences. Fission yeast ( *Schizosaccharomyces pombe* ) is a rod-shaped ascomycete fungus in which cell cycle progression is linked to changes in cell length. Microscopy work to obtain these metrics places considerable burdens on time and effort. We now report on Photo Phenosizer (PP), a machine learning-based methodology that measures cell dimensions in fission yeast. It does this in an unbiased, automated manner and streamlines workflow from image acquisition to statistical analysis. Using this new approach, we constructed an efficient and flexible pipeline for experiments involving different growth media (YES and EMM) and treatments (Untreated and MMS) as well as different genotypes ( *cut6-621* versus wildtype). This methodology allows for the analysis of larger sample sizes and faster image processing relative to manual segmentation. Our findings suggest that researchers using PP can quickly and efficiently determine cell size differences under various conditions that highlight genetic or environmental disruptions.

**Figure 1. A rapid machine-learning method to segment and measure cells:**
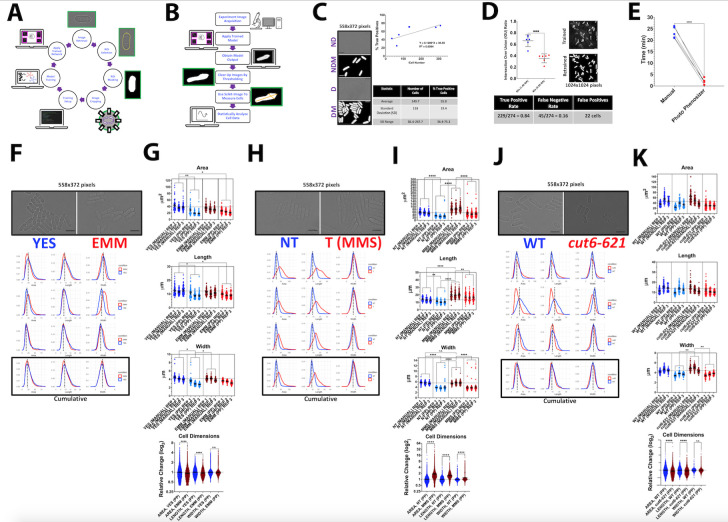
**A** . Neural training to generate image weights output. The training process consists of obtaining microscope images of fission yeast and creating a dataset of the original images and mask equivalences for the training session input. Training resulted in a weights file that contains all the information on what the program will be looking for when given an unseen microscope image of fission yeast cells. **B.** Application of trained weights output on experimental images. The program takes in a trained weights file that comes from the training process in ( **A** ) and uses it to segment fission yeast cells by creating a mask image. The mask image is then cleared up downstream by various kinds of functions such as erosions and dilations that threshold, fill in any holes in the cells, and attempts to separate the touching cells. The dimensions are then measured for each cell with measurement functions and then written into a comma-separated values file (csv) that can then be further processed for statistical analysis in R. **C.** Cell densities influence the performance of the program. There is a positive correlation between the number of cells and true positive rates. D: Dense; ND: Not Dense; DM: Dense Mask; NDM: Not Dense Mask. The size of all the images shown is 558 x 372 pixels (70.20 x 46.80 micrometers) and the size of the image area where cells are counted is 1920 x 1440 pixels (241.56 x 181.17 micrometers). GraphPad Prism was used to create scatterplots and to provide descriptive statistics. **D.** IOUs were determined between two different magnifications, 40x and 60x. True positives, false negatives, and false positives rates were also calculated. PP was first trained with eleven 60x images acquired in a Keyence BZ-X800 microscope system. Retraining of PP was carried out with six 60x images obtained in a DeltaVision Microscope. GraphPad Prism was used to create the box plot and test for significance using a two-tailed, unpaired t-test. The asterisks (***) represent a p value < 0.001. **E.** The efficiency of PP to segment and statistically analyze 100 cells was compared to manual segmentation. Five individuals recorded the time it takes them to manually segment and statistically analyze cell dimensions and their results were compared to the time it takes them to run PP with the same pictures. GraphPad Prism was used to create the plot and to test for significance using a two-tailed, paired t-test. The asterisks (****) represent a p value < 0.0001. **F.** Density plots and brightfield images show the phenotypes differences between cells treated with YES and those treated with EMM. The scale bars shown in the micrographs indicate 10 µm in length. **G.** PP was implemented to compare wildtype fission yeast grown in YES (trials=3, biological replicates=3 per trial, total cells=4668) and EMM plus supplements (HULA) media (trials=3, biological replicates=3 per trial, cells=4812) to observe the differences in cell dimensions by density and box plots. Dotted lines in all density plots show the peak of baseline profiles in each experiment. A sample of cells processed by PP were randomly selected among three trials per medium and segmented manually to compare method outcomes. The box plots show data for cells grown in different media (YES cells=217, EMM cells=218). GraphPad Prism was used to create box plots and to test for significance using a two-tailed, unpaired t-test for cumulative data and a nested one-way ANOVA followed by Šídák's test for multiple method comparisons. All density plots were statistically analyzed using a two-sample Kolmogorov-Smirnov test in R to test for differences in cell dimensions distributions. The asterisks (*, **, and ****) correspondingly represent p values < 0.05, 0.01, and 0.0001. Brackets show pairwise comparisons with significant differences. Lack of brackets indicates no significance. The box plot error lines represent the average +/- 95% confidence intervals (CI), respectively. **H.** Density plots and brightfield images show the phenotypes differences between cells treated with MMS (T) and those treated with nothing (NT). **I.** PP processed images of cells grown in YES (trials=3, biological replicates=3 per trial, cells=7966) and in YES plus MMS (trials=3, biological replicates=3 per trial, cells=5285). Comparison of PP to manual segmentation is carried out as in panel ( **G)** (YES cells=208, YES+MMS cells=208). GraphPad Prism was used to create box plots and to test for significance using a two-tailed, unpaired t-test for cumulative data and a nested one-way ANOVA followed by Šídák's test for multiple method comparisons *(i.e.,* manual vs pp). The asterisks (**, ***, and ****) represent p values < 0.01, 0.001, and 0.0001, respectively. Brackets show pairwise comparisons with significant differences. For omitted significant pairwise comparisons, see the Extended Summary Statistics file included. The box plot error lines represent the average +/- 95% confidence intervals (CI), respectively. The scale bars shown in the micrographs indicate 10 µm in length. **J.** Density plots and brightfield images show the phenotypes differences between a lipid metabolism mutant, *cut6-621* (MP218) and the wildtype strain (JB32). The scale bars shown in the micrographs indicate 10 µm in length. **K.** PP was employed to compare the *cut6-621* (trials=3, biological replicates=3 per trial, total cells=4529) and the wildtype strains (trials=3, biological replicates=3 per trial, cells=2612) to observe the differences in cell dimensions by density and box plots. A sample of cells processed by PP were randomly selected among three trials per genotype and segmented manually to compare method outcomes. The box plots show data for cells of different genotypes ( *cut6-621* cells=217, WT (JB32) cells=218). GraphPad Prism was used to create box plots and to test for significance using a two-tailed, unpaired t-test for cumulative data and a nested one-way ANOVA followed by Šídák's test for multiple method comparisons. The asterisks (** and ****) correspondingly represent p values < 0.01 and <0.0001. Brackets show pairwise comparisons with significant differences. Lack of brackets indicates no significance. The dotted and solid error lines represent the average +/- 95% CI, respectively.

## Description

*Cell dimensions and the cell cycle*

The dimensions of a cell are linked to its life stages and environmental conditions (Molenaar *et al.* 2009). Homeostatic control dictates cellular growth and division, placing species-specific limits on cell size (Jun and Taheri-Araghi 2015). In eukaryotes, whether cells employ strategies that monitor changes in size, time of growth, or volume expansion, the cell cycle governs when nuclear and cellular separation are executed (Facchetti *et al.* 2017; Cadart *et al.* 2019; Zatulovskiy and Skotheim 2020). Therefore, much can be learned about the conditions that affect important cellular functions by using microscopy to examine relevant cell metrics.

Fission yeast is a rod-shaped ascomycete fungus in which cell cycle progression can be closely tracked by changes in cell length (Nurse 1975; Nurse *et al.* 1976). As its common name implies, fission yeast divides by medial fission and grows, predominantly using a sizer strategy (Facchetti *et al.* 2017) to an average length of 14 µm before division (Nurse 1975; Fantes 1977; Mitchison and Nurse 1985; Facchetti *et al* . 2019). In this organism, cellular septation and cytokinesis coincide with S-phase.

Consequently, progression through the cell cycle in wildtype cells can be inferred by following changes in length; from shortest in late-S/early-G2 to longest in late-M/early-S (Nurse *et al.* 1976; Mitchison and Nurse 1985; Martin 2009). Observations into mutants that alter area and length have substantially expanded our understanding of the cell cycle and its connection to processes that regulate homeostatic control of cell size (Nurse 1975; Nurse *et al.* 1976; Moseley *et al.* 2009; Navarro and Nurse 2012; Facchetti *et al* . 2019; Scotchman *et al.* 2021).

*Microscopy bottleneck*

Although microscopic examination of fission yeast allows for quick determination of life cycle status under different genetic and environmental conditions, the task of measuring and subsequently analyzing size metrics statistically places considerable burdens on time and effort (Rallis and Bähler 2016). The speed of automated microscope image acquisition and processing is often stifled by the bottleneck of parsing through and individually selecting regions of interest (ROI) to measure cell dimensions. Moreover, outlining cells either manually or using plugin packages for commonly used image analysis software (Schindelin *et al.* 2012) results in data affected by user bias or processing artifacts (Chessel and Carazo Salas 2019).

*Image processing* *automation*

We report a machine learning-based pipeline, Photo Phenosizer (PP), that measures cell dimensions in fission yeast. This approach involves training an artificial neural network, using the network to create approximate image masks which predict the location of cells within experimental bright field images, and using image processing functions to sharpen the masks and measure the cell dimensions.

PP begins with an artificial neural network that is trained using microscopy images paired with manually annotated regions of interests (ROIs) **(Figure 1A** ) **.** These are represented as binary mask images which use white pixels to represent the ROIs and black pixels to represent the background. To predict ROIs for unannotated images, the trained neural network outputs a grayscale mask in which black pixels indicate strong confidence that the original pixel is part of the background. White pixels indicate strong confidence that the original pixel is part of a cell, and gray pixels indicate varying levels of confidence.

We then use thresholding to turn this grayscale mask into a binary mask by setting each pixel to black or white depending on whether it is below or above a certain threshold. After thresholding, we apply a series of erosion and dilation operations to disconnect adjacent cells, fill in holes, and remove small artifacts. Next, isolated regions of white are identified and those whose areas fall below a certain threshold are removed. We then calculate the length and width of the remaining cell regions by finding their maximum and minimum Feret diameters, respectively ( **Figure 1B** ).

We processed image data from multiple experiments in a time-efficient manner without the need for extensive computing power. To these processes, we coupled a downstream statistical routine that is simple to implement and from which relevant cell growth characteristics of large statistical samples can be interpreted ( **Figure 1A-K** ).

*Efficiency and fidelity of cell dimension measurements*

Our rationale for employing an automated approach to image processing was to increase efficiency in data acquisition and to decrease user bias. We tested the ability of PP to identify and select single cells among monolayer clusters. We found that cell segmentation correlates positively with increasing number of cells **(Figure 1C).** Between 32 and 268 cells are needed per field of view for PP to achieve segmentation true positive rates of 0.36-0.75. Rotational dispersion of monolayers as previously reported (Escorcia *et al.* 2019) ensured that PP unbiasedly captured a large proportion of cells in each field of view.

To assess the accuracy of PP’s automated segmentation, we calculated the intersection over union (IOU) ratios between masks produced by manual and automated segmentation (Yu *et al.* 2016) **(Figure 1D** ) **.** The mean IOU ratio was between 0.56-0.76 (standard deviation range), which is reasonable for image processing at this level but warrants further optimization for improved accuracy. In this training context, PP positively identified cells at a rate of 0.84 **(Figure 1D** ). To determine PP’s accuracy on a different dataset, we processed images taken at 40x magnification and compared them to segmentation carried out at 60x ( **Figure 1D** ). We observed a 31% decrease in accuracy, which indicates PP must be retrained to accommodate different magnifications.

To confirm that retraining of PP increases its segmentation accuracy, we used 60x images of fission yeast cells grown in a different lab and acquired by a different microscope system. After 25 hours of retraining, PP was able to effectively detect and segment cells in images obtained by two different microscopes ( **Figure 1D** ). This implies that researchers who use PP will likewise have to carry out additional training of the software to adapt to their own imaging systems. Therefore, these data suggest that under appropriate cell densities and microscope-specific training, PP can identify and measure the dimensions of hundreds to thousands of fission yeast cells in a relatively short period of time.

Image processing time is a limiting factor in most microscopy experiments. Thus, we tested if PP was faster than five people using Fiji and RStudio to correspondingly create measurable ROIs and statistically analyze cell dimensions (Schindelin *et al.* 2012; Allaire 2012) ( **Figure 1E** ). We carried out this test on different operating systems and at various levels of computational power. We observed a consistent, ten-fold decrease in processing time using PP relative to manual efforts ( **Figure 1E** ). This indicates that PP can be integrated as a rapid, unbiased tool in microscopy workflows to increase experimental sample sizes and thus robustness of downstream statistical analyses.

*Cell dimensions following growth in different media*

The environment in which cells grow influences progression through the cell cycle (Martin 2009). In fission yeast, cell dimensions like area and length provide useful information regarding vegetative growth and cellular stress (Mitchison and Nurse 1985). We asked if PP was sensitive enough to capture cell dimension differences in response to rich (YES) and supplemented minimal media (EMM+HULA), which are difficult to discern by visual inspection alone ( **Figure 1F** ) **.** Although we observed moderate variation in cell metric distributions across three experiments, the cumulative density plots reveal that cells grown in EMM are shifted toward the lower end of each cell dimension distribution ( **Figure 1F** ).

To assess the accuracy of PP in generating these results, we compared its outcomes to those obtained by hand on a randomly selected group of cells. The results show that while manual segmentation provides exact cell measurements, PP generates approximate dimension values that are 42.6% (area), 22.8% (length), and 26.7% (width) lower than manual outlining of YES grown cells ( **Figure 1G** ). For cells cultured in EMM, PP’s performance relative to manual segmentation is also lower by 27.5% (area), 13.4% (length), and 15.8% (width) ( **Figure 1G** ). Both methods showed no significant differences in cell dimensions between cells grown in YES or EMM **(Figure 1G; Extended Summary Statistics** ) **.** These data indicate that although PP underestimates exact measurements due to segmentation thresholding that allows for separation of cells in a cluster, at present, it can be used to make useful relative comparisons of cell dimension approximations.

When we used PP to examine the relative cumulative approximations of cells grown in different media, we observed that growth in YES results in cells that are 8.2% larger and 6.6% longer than cells grown in EMM ( **Figure 1G** ). Though these variations are small, they are consistent with metrics observed when cells are grown in rich versus minimal media (Kelly and Nurse 2011; Zach *et al* . 2018; Patterson *et al* . 2019; Facchetti *et al.* 2019). Factors that communicate stress states to cells influence pathways that regulate the cell cycle and thus cell dimensions (Nurse and Nasmith 1976; Kelly and Nurse 2011; Pan *et al* . 2014; Chica *et al.* 2016). As cell growth progresses, EMM decreases in pH faster than YES resulting in cells with slower doubling times. This phenotype difference is facilitated by Cbf11, a transcription factor involved in cell cycle regulation (Zach *et al* . 2018). Similarly, disruption of mitotic entry by genetic or environmental elements is associated with cell cycle mutants that affect cell length and related metrics (Moseley and Nurse 2009; Patterson *et al.* 2019). Thus, the relative differences we observed in area and length may be associated with prolonged duration of G2 in cells exposed to elements in EMM that disrupt progression to M/G1-S.

To observe how well PP captures phenotype differences resulting from the response to cellular stress, we exposed wildtype cells to sublethal doses of methyl methanesulfonate (MMS), which promotes DNA alkylation damage and thus disrupts cell cycle progression (Ranatunga and Forsburg 2016; Willis *et al* . 2016). We observed that relative to untreated samples, cells exposed to MMS exhibit cell density profiles that are shifted toward the higher end of cell dimension distributions ( **Figure 1H** ). Although PP segmentation only approximates cell measurements and underperforms relative to manual tracing, its results are consistent with the trends observed previously with cells grown in rich versus minimal media. The comparison between manual and PP segmentation in distinguishing cell dimension differences resulting from genotoxic exposure reveals that area and length but not width changed in response to this cellular stress ( **Figure 1I; Extended Summary Statistics** ) **.** Thus, we proceeded to examine the relative change in cell metrics of cells experiencing genotoxicity. A large proportion of MMS-treated samples show cells that are 84% larger, 56% longer, and 18.3% wider than their untreated counterparts ( **Figure 1I** ). This effect is consistent with the morphological impacts of the G1/S checkpoint following MMS exposure. DNA damage repair results in cell elongation and enlargement of most cells in G2, while cell cycle resumption is linked to short and small cells (Ranatunga and Forsburg 2016; Willis *et al* . 2016) ( **Figure 1I** ) **.** These two important cell dimension changes are effectively captured by PP, indicating that it can be used to examine cell cycle-related phenotypes. All in all, these data suggest that PP can be employed as a quick diagnostic tool to ascertain subtle morphological differences of cultures grown in different conditions and treatments.

*Cell dimensions in a mutant with disrupted lipid metabolism*

Lipid metabolism is essential for cell signaling, energetics, and growth (Chung 2021). Disruption of lipid homeostatic control often results in abnormal cell phenotypes (Olzmann and Carvalho 2019). We reasoned that if we used PP to interrogate a lipid regulator mutant ( *cut6-621* ), we would observe cell dimensions linked to deregulated growth stemming from impaired lipid dynamics. Consistent with this hypothesis and relative to the wildtype strain, the density plots of the *cut6-621* mutant show moderate shifts toward the lower end of cell dimension distributions ( **Figure 1J** ). Moreover, PP and manual segmentation of these strain pairs is comparable. However, this may result from high variance in the metrics measured by both methods rather than from enhanced PP performance **(Figure 1J** ) **.** Both methods showed no significant differences in cell dimensions between the wildtype and *cut6-621* strains **(Figure 1J; Extended Summary Statistics** ). Upon close inspection of the relative change in metrics of these cells, we observed that compared to the wildtype strain, the *cut6-621* mutant exhibits cells that are 8.8% smaller and 7.7% shorter during logarithmic growth ( **Figure 1K** ).

These observations coincide with the *cut* phenotype reported for these cells by Převorovský and colleagues (Zach *et al.* 2018), but caution is warranted regarding the biological significance of this. In the experimental conditions of this study, phenotype differences relative to the wildtype were only moderately significant. It is possible that examining cell dimensions differences is best observed in media with different nitrogen sources as previously reported (Zach *et al.* 2018). Since Cut6 is involved in the regulation of lipid storage and metabolism, which are crucial for progression through the cell cycle, its disruption in the *cut6-621* mutant results in chromosome mis-segregation due to premature septation. This leads to cells with abnormal cell dimensions and growth dynamics, especially if exposed to a rich medium (Zach *et al* . 2018). Indeed, we observed that *cut6-621* cells show a diversity of pseudo-hyphal, septated, and short-cell morphologies as compared to the wildtype strain ( **Figure 1K** ). These results, therefore, indicate that PP can be optimized in appropriate growth conditions to distinguish cell dimension differences stemming from genotypes that affect growth dynamics and cell cycle progression.

*Research significance of PP*

Our findings suggest that PP will streamline fission yeast microscopy observations. The increased efficiency in processing time will enable examination of large sample sizes that reveal robust cell phenotypes with relevant biological and statistical significance. Moreover, the overall versatility of this machine-learning approach will increase the potential of PP based on the needs of each researcher. The prospect of going from image acquisition to statistical analysis in less than ten minutes will decrease the frustration and increase productivity and overall happiness of researchers handling fission yeast cultures.

*Conclusion*

PP is a pipeline that will enable researchers to examine growth dynamics, genotype differences that affect cell dimensions, and the effects of different environmental stressors. The pipeline automates cell segmentation and cell measurements and facilitates statistical analysis of the results. While PP decreases image processing time relative to manual segmentation, in its current version, it is microscope-specific thereby requiring additional training to adapt to different microscope settings. However, researchers can train PP for their purposes with only a few additional annotated images. Moreover, the authors will provide access to the trained model used in this study upon request. As the community of fission yeast researchers begins to use PP, we expect the segmentation algorithm to improve by additional training. Finally, we look forward to collaborating with other research groups to enhance features that are specific to microscopy work in fission yeast.

## Methods

*Computational Methods*

All software was implemented using the Python programming language. We used the DeepLabv3 artificial neural network architecture to train our model (Chen *et al.* 2017). To reduce training times and the required amount of training data, we used transfer learning which began with the deeplabv3_resnet101 pretrained model from the PyTorch hub (Paszke *et al* . 2019). The DeepLabv3 FineTuning software was then used to further train the model (Minhas 2019). Eleven 1920x1440 micrographs containing anywhere from 24-272 cells per image were used to train the model. Training data was augmented using cropping and rotation to provide more input data for training purposes, which we found yielded better segmentation results. Both a MacBook Pro 16 with the M1 Pro Processor and 32 GB of RAM and a Windows Desktop PC with a 6 core AMD Ryzen Processor and 16 GB of RAM were used in the training process. Training took approximately 4-6 hours on both machines.

The OpenCV library was used for thresholding, erosion and dilation (Bradski 2000). We experimented with different parameter values for these three methods. Thresholding was performed with a threshold value of 170. Numerous erosion and dilation operations were carried out using a 3x3 kernel size and each operation involved 2-4 iterations. Cell identification and area measurements were carried out using the scikit-image library (Stéfan *et al* . 2014). The Feret library was used to calculate the maximum Feret diameter and the minimum Feret diameter for length and width respectively (Nwt 2022).

PP’s accuracy was assessed using a set of microscopy images that were not included in the training set. For this evaluation set, we produced manually generated and PP generated masks and for each pair calculated the Intersection Over Union (IOU) ratio and the number of true positives, false negatives, and false positives.

To calculate the IOU for a pair of masks in the evaluation set, the Area of Intersection (AOI) was computed by counting the number of pixels that were white in both masks, and the Area of Union (AOU) was computed by counting the number of pixels that were white in either mask. Finally, the IOU was calculated by dividing the AOI by the AOU.

The number of true positives, false positives, and false negatives for each pair of masks was calculated using scikit-image to identify the cell regions of each mask. For each region in the PP mask, we identified its centroid and if the pixel at the centroid coordinates in the manual mask was white, we recorded a true positive, otherwise we recorded a false positive. False negatives were similarly identified using the cell centroids of the manual mask.

All software and documentation can be found on GitHub ( https://github.com/XavierCompBio/PhotoPhenosizer ). Contact the authors for access to the trained model.

*Statistical Analysis*

R Studio and R were used for all statistical analyses (Allaire 2012; R Computing Team 2013), while GraphPad Prism was employed to graph time and cell metrics box plots and to calculate descriptive statistics (GraphPad Software, San Diego, CA). Scatterplots and Pearson’s correlation were used to relate area, length, and width measurements in identified cells across all experiments. Kernel density estimation (density plot) was used to provide a visual comparison of cell measurements across different experimental conditions. Statistical differences in the mean, 75 ^th^ percentile (Q3), and 90 ^th^ percentile cell measurements across experimental conditions were evaluated using t-tests and quantile regression. Variability was analyzed using the coefficient of variation (CV), and skewness was assessed via the third moment of the data distribution found using the “moments” R package (Komsta and Novomestky 2015). For the lengths of cells showing biphasic histograms, we applied a two-component mixture model with mixing proportions and parameters estimated via an EM algorithm as implemented in the “mixtools” R package (Benaglia *et al.* 2009).

The R scripts that were used in this study are freely available on the GitHub repository mentioned above. The method originated as an application of transfer learning from existing image processing neural networks, and we encourage others to adopt it within this framework. In this paper, we demonstrated the viability of such an approach using data from our lab. In other lab settings, some additional training may be necessary to achieve comparable performance; however, this is easily achieved by modifying and rerunning the available Python files. Our motivation was not to present an out-of-box tool that is ready to be applied in all circumstances, but rather a useful application of transfer learning that others can build off to enhance the productivity of their own experiments.

*Cell Growth and Culture*

Standard techniques, culture conditions, and media were used as previously described (Sabatinos and Forsburg 2010). Yeast extract with supplements (YES 225, 2011-300; Sunrise Science Products) was employed for all experiments involving liquid medium, except for cultures grown in Edinburgh minimal medium (EMM, 2005-500ML; Sunrise Science Products) supplemented with 225 mg/l each of L-histidine (ICN10195480; Fisher Scientific), uracil (U0013100G; Fisher Scientific), L-leucine (AAA1231122; Fisher Scientific), and adenine hemisulfate (321-30-2; Millipore Sigma). For solid medium, bacteriological agar (Ultrapure, AAJ10906P5; Thermo Scientific) was added to liquid YES and poured into 100x15 mm polystyrene petri dishes (8609-0010; USA Scientific). A *Schizosaccharomyces pombe* strain derived from 972 h- (FY527) and used in a previous report (Escorcia and Forsburg 2017) was employed in all YES versus EMM and in untreated (NT) versus treated (T, 0.01% MMS) experiments. All MMS work was done as previously carried out in (Ranatunga and Forsburg 2016). Wildtype (JB32) and *cut6-621* (MP218) strains (Zach *et al* . 2018) used in genotype experiments were kindly gifted to us by Martin Převorovský. A 3 ml YES starter was used to subculture cells that were subsequently grown in 5 ml YES inside an incubator shaker at 32 ^o^ C to OD _abs595_ 0.3-0.6. Samples were then harvested and washed thrice in YES or EMM-HULA before being processed for microscope acquisition as reported in (Escorcia *et al.* 2019).

*Bright field Microscope Image Acquisition*

Microscope slides containing fission yeast samples were prepared as detailed in (Escorcia *et al.* 2019). Briefly, 1 ml samples were precipitated by centrifugation. Cell pellets were resuspended in 100 µl YES or EMM-HULA and 10 µl of sample spotted onto 2% agarose (BP160-100; Fisher Scientific) gel pads placed atop glass microscope slides (16004-422; VWR). A coverslip (16004-302; VWR) was put on top of the sample and rotated clockwise (rotational dispersion) twice to create an evenly distributed cell monolayer. To seal the coverslip in place, 1:1:1 (w/w) molten Vaseline:lanolin:paraffin (VaLaP) solution was applied at the edges with a wooden stick. The slide was allowed to equilibrate to room temperature for 15 minutes before image acquisition. Samples used for imaging were derived from 3 trials consisting of 3 biological replicates. Five fields of view were captured per biological replicate. At least 30 cells were imaged per field of view.

Imaging was carried out as described in (Escorcia *et al.* 2017; Escorcia *et al.* 2019; Escorcia *et al.* 2021) with a few changes. Cells were staged at room temperature (~22 ^o^ C) using an inverted BZ-X800E Keyence microscope equipped with a 40x (0.95 NA) and 60x (1.40 NA) oil immersion Plan Apochromat objective lens (BZ-PA60), 3.7 W LED light source, a 2.3 million pixel, 8-bit monochrome charged-coupled device (CCD) camera, and BZ-H4A BZ-X800E Analyzer software. Images were saved as TIF files for downstream processing of data. Cells imaged outside our lab were acquired using similar experimental conditions and a DeltaVision microscope with softWorRx version 4.1 (GE Healthcare; Issaquah, WA) equipped with a 60×/ 1.4 NA Plan-Apo lens, solid-state illuminator, and 12-bit charge-coupled device (CCD) camera.

## Reagents

**Table:** 

**Reagent (catalogue number)**	**Vendor**
YES 225 (2011-300)	Sunrise Science Products
Edinburgh minimal medium (2005-500ML)	Sunrise Science Products
L-histidine (ICN10195480)	Fisher Scientific
Uracil (U0013100G)	Fisher Scientific
L-leucine (AAA1231122)	Fisher Scientific
Adenine hemisulfate (321-30-2)	Millipore Sigma

## Extended Data

Description: Link to the Github. Resource Type: InteractiveResource. DOI: 10.22002/D1.20253

Description: Descriptive and Summary Statistics. Resource Type: Dataset. DOI: 10.22002/D1.20251
